# Interaction prediction and classification of PDZ domains

**DOI:** 10.1186/1471-2105-11-357

**Published:** 2010-06-30

**Authors:** Sibel Kalyoncu, Ozlem Keskin, Attila Gursoy

**Affiliations:** 1 Center for Computational Biology and Bioinformatics, College of Engineering, Koc University, Rumelifeneri Yolu 34450 Sariyer, Istanbul, Turkey

## Abstract

**Background:**

PDZ domain is a well-conserved, structural protein domain found in hundreds of signaling proteins that are otherwise unrelated. PDZ domains can bind to the C-terminal peptides of different proteins and act as glue, clustering different protein complexes together, targeting specific proteins and routing these proteins in signaling pathways. These domains are classified into classes I, II and III, depending on their binding partners and the nature of bonds formed. Binding specificities of PDZ domains are very crucial in order to understand the complexity of signaling pathways. It is still an open question how these domains recognize and bind their partners.

**Results:**

The focus of the current study is two folds: 1) predicting to which peptides a PDZ domain will bind and 2) classification of PDZ domains, as Class I, II or I-II, given the primary sequences of the PDZ domains. Trigram and bigram amino acid frequencies are used as features in machine learning methods. Using 85 PDZ domains and 181 peptides, our model reaches high prediction accuracy (91.4%) for binary interaction prediction which outperforms previously investigated similar methods. Also, we can predict classes of PDZ domains with an accuracy of 90.7%. We propose three critical amino acid sequence motifs that could have important roles on specificity pattern of PDZ domains.

**Conclusions:**

Our model on PDZ interaction dataset shows that our approach produces encouraging results. The method can be further used as a virtual screening technique to reduce the search space for putative candidate target proteins and drug-like molecules of PDZ domains.

## Background

Protein-protein interactions play fundamental roles in signal transduction, formation of functional protein complexes and protein modification [1]. One of the most common protein interaction domains in the cell is PDZ domain which is a central signaling protein of most species [2-4]. The PDZ domains, among other nearly 70 distinct recognition domains, are crucial because they are involved in development of multi-cellular organisms by constructing cell polarity, coordination of intercellular signaling system and directing the specificity of signaling proteins [5]. They consist of 80 to 90 amino acids and have a compact globular fold composed of a core of six β strands (βA - βF) and two α helices (αA, αB). By binding the C-terminal motifs of their target proteins, PDZ domains target, cluster and route these proteins [6]. However, some PDZ domains also can bind to the internal motifs of target proteins, lipids and other PDZ domains [3,7].

**Figure 1:**
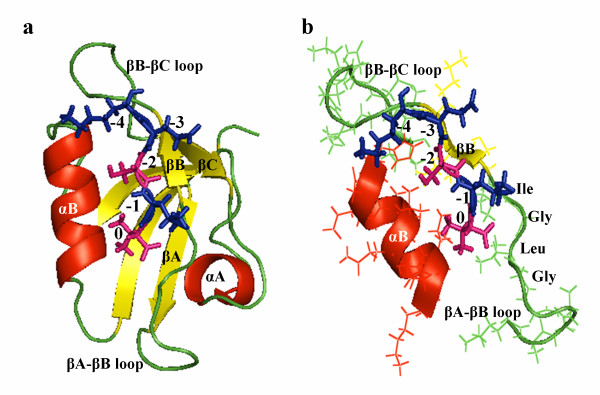
**Representative structure of a PDZ domain in complex with its ligand**. (a) The common representation of a PDZ domain (α-1 syntrophin) with a peptide (in its stick form) in its binding pocket. Peptide positions -1 and -3 (blue) point towards to the solvent, the positions 0 and -2 (pink) head towards to the binding pocket (b) The interaction of the peptide with αB helix and conserved GLGF segment (here it is GLGI) of the βA-βB loop (PDB ID:2PDZ).

C-terminus of a peptide recognizes and binds to a pocket between carboxylate-binding loop (βA - βB loop) that contains the conserved GLGF motif, and αB helix of the PDZ domain [8-11], this is also called the canonical binding. The ligand binds to the PDZ domain as an anti-parallel extension of the β-sheet of the domain and while ligand positions -1 and -3 head towards to the solvent, the positions 0 and -2 point towards to the binding pocket [12] (Figure 1). Therefore, it can be suggested that ligand positions 0 and -2 are very crucial for recognition and binding to target proteins. The importance of these two positions also lead to the general classification of PDZ domains into three classes according to short peptide motifs of the last three residues at the extreme C-termini of their peptide ligands. Class I PDZ domains bind to C terminal motifs with the sequence of [Ser/Thr-X-Φ COOH], Class II PDZs bind to the sequence of [Φ-X-Φ-COOH] and Class III PDZs prefer the sequence of [Asp/Glu-X-Φ-COOH] where Φ is any hydrophobic amino acid and X is any amino acid. However, some PDZ domain interactions do not satisfy these restrictive types of recognition and so additional classes and additional important residues are proposed to exist for ligand specificity of PDZ domains [12-16]. For example, Songyang *et al. *investigated the binding specificities of nine PDZ domains by using an oriented peptide library and concluded that additional selection specificities, depending on up to -8 position of the peptide ligand, were observed beside the 0 and -2 positions [17].

Although PDZ domains show selectivity toward their target ligands, they also display promiscuity, binding to more than one ligand, and degenerate specificity [18-21], so interaction prediction of these domains can be challenging. Several studies aimed to classify and predict interaction specificity of PDZ domains that could save time-consuming and expensive experiments. Chen *et al. *[22] predicted PDZ domain-peptide interactions from primary sequences of PDZ domains and peptides by using a statistical model and reported an area under curve (AUC) value of 0.87 for extrapolations to both novel mouse peptides and PDZ domains. Bezprozvanny and Maximov [15] used a classification method based on the two critical positions of 249 PDZ domains and they presented 25 different classes of PDZ domains. Stiffler *et al. *[23] tried to characterize the binding selectivity of PDZ domains by training multi-domain selectivity model for 157 mouse PDZ domains with respect to 217 peptides and they indicated that PDZ domains are distributed throughout the selectivity space contrary to discrete specificity classes. Schillinger *et al. *[24] used a new approach, Domain Interaction Footprint (DIF), to predict binding peptides of SH3 and PDZ domains by using only the sequence of the peptides and they reported an AUC value of 0.89 for PDZ multi-domain model by using the sequence information of binding and non-binding peptides of four different PDZ domains. Tonikian *et al. *[25] constructed a specificity map consisting of 16 unique specificity classes for 72 PDZ domains and this lead to the prediction of PDZ domain interactions. Wiedemann *et al. *[21] tried to quantify specificity of three PDZ domains by relating the last four C-terminal motifs of their ligands to the corresponding dissociation constants which can provide selectivity pattern of PDZ domains and design of super-binding peptides. Eo *et al. *[26] used an SVM classifier by adapting amino acid contact matrices and physiochemical distance matrix as a feature encoding in order to identify PDZ domain ligand interactions.

In this study, we propose a method to predict PDZ domain-peptide interactions by using only the sequence information of PDZ domains and ligands. In order to construct a numerical feature vector for each interaction, trigram and bigram frequencies of each primary sequence of PDZ domains and peptides are calculated. We obtain a high prediction performance (accuracy of 91.4% and AUC of 0.97 for trigram model) distinguishing between binding and non-binding peptides of PDZ domains. We make use of the most commonly used classifiers (SVM, Nearest Neighbor, Naïve Bayes, J48, Random Forest) and find Random Forest classifier with the best prediction accuracy. Moreover, we show that our method can be efficiently used to distinguish between Class I, Class II and Class I-II PDZs (both binding to Class I and Class II peptides) with an accuracy of 90.7% and AUC of 0.90 for trigram model.

## Methods

### Dataset

For interaction prediction part, a positive (binding) and a negative (non-binding) dataset are needed in our machine learning model. The PDZ interaction dataset is retrieved from the study of Stiffler *et al.*, which is composed of interaction data of 85 mouse PDZ domains with respect to 217 mouse genome-encoded peptides [23,24]. They used the combination of protein microarrays and fluorescence polarization (FP) methods to identify biological interactions of PDZ domains. In the current study, only binding and non-binding information that were confirmed by FP is used as the training set due to the fidelity of FP. After selection of FP confirmed interactions, we obtained 731 binding and 1361 non-binding interactions between 85 PDZ domains and 181 peptides (See additional file 1: Table S1 for PDZ interaction data).

An independent validation dataset is also used in interaction prediction part in order to test the predictive performance of our model. The validation dataset is extracted from the previous study of Stiffler *et al. *and it is composed of 27 binding and 62 non-binding interactions of 16 PDZ domains and 20 peptides [27] (See additional file 2: Table S2 for validation interaction data).

For class prediction part, 86 PDZ domains are categorized, resulting in 45 Class I, 20 Class II, 21 Class I-II. These are retrieved from our interaction dataset and PDZBase [28] by looking at their interactions with different classes of peptides. PDZ domains are annotated as Class I and Class II according to the C terminus sequence of the interacting peptides, [Ser/Thr-X-Φ-COOH] for Class I peptides and [Φ-X-Φ-COOH] for Class II peptides, respectively. Class I-II PDZ domains are determined if they bind to both Class I and Class II peptides. (See additional file 2: Table S3 for class data).

In order to be consistent in our interaction prediction model, we took the last 10 residues of each peptide sequence due to the selection specificities of PDZ domains up to -10 positions of peptides. The sequence data of PDZ domains and peptides can be seen in additional file 2: Table S4 and Table S5, respectively.

### Feature encoding

Frequencies of consecutive three amino acids (trigram) and two amino acids (bigram) in the primary sequences are used as features. For instance, a sequence of "ABCDE" results in a trigram set of "ABC", "BCD", "CDE" and a bigram set of "AB", "BC", "CD" and "DE". In order to reduce the dimension of the features, 20 amino acids are clustered into 7 different classes (Table 1) according to their dipoles and volumes of the side chains which reflect their interaction specificity by giving an insight about their electrostatic and hydrophobic natures [29].

**Table 1:** Seven amino acid classes used in our model.

Class	Amino acid(s)	Volume (Å^3^)	Dipole (Debye)
1	Ala, Gly, Val	<50	0
2	Ile, Leu, Phe, Pro	>50	0
3	Tyr, Met, Thr, Ser	>50	<1.0
4	His, Asn, Gln, Trp	>50	1.0 < Dip. < 2.0
5	Arg, Lys	>50	2.0 < Dip. < 3.0
6	Asp, Glu	>50	>3.0
7	Cys*	>50	<1.0

*Cys is differentiated from class 3 because it can form disulfide bonds

To calculate trigram frequency of the PDZ and corresponding peptide sequences, the number of occurrence of each subsequent trigram in the sequence is counted, and this number is divided by the total number of trigrams in the sequence which is (n-2), where n is the sequence length. At the end, we obtain 343 (7 × 7 × 7) features for each sequence because amino acids are clustered into seven classes resulting in 7 × 7 × 7 different combination of trigrams. For the interaction prediction part, feature vector space is constructed by combining trigram frequency sets of both PDZ domain and corresponding peptide which gave 686 features for each interaction (343 for PDZ domain, 343 for peptide). For bigram frequency calculation, the same procedure is applied and we obtained 49 (7 × 7) features for each sequence and a total of 98 features (49 for PDZ domain, 49 for peptide) are constructed for each interaction. Therefore, we constructed a feature vector space (X, Y, W) to represent an interaction:

Here, X is the feature vector space of the PDZ sequence, and each feature x_i _represents the frequency of each trigram where i = 1, 2,...., 343 or each bigram where i = 1, 2,..., 49, Y is the feature vector space of peptide sequence, each feature y_i _represents the frequency of each trigram or bigram, and W is the corresponding label that contains binary data (w_1_: binding, w_2_: non-binding). Thus, a 686 dimensional vector for trigram part and a 98 dimensional vector for bigram part are constructed to represent each binding/non-binding interaction.

For the class prediction part, the peptide sequences are discarded and only the sequences of PDZ domains are used to construct the feature vector space, because peptide sequences are used as the label of the dataset. Therefore, a 343 dimensional vector space for trigram part and 49 for bigram part with three labels (w_1_: ClassI, w_2_: ClassII, w_3_: ClassI-II) are built to represent each class of PDZ domains.

### Model comparison

There are several machine learning approaches to predict domain interactions [30-32]. We chose five classifiers, SVM (Support Vector Machine), Nearest Neighbor, Naïve Bayes, J48 and Random Forest which have been commonly used in protein-protein interaction prediction problems. In SVM algorithm, feature vectors are non-linearly mapped on a high dimensional feature space and a set of hyperplanes are constructed to be used for classification or regression [33]. The simplest one among used classifiers is Nearest Neighbor which classifies instances according to their closeness to the training examples [34]. The basic idea behind Naïve Bayes is to predict the class of an instance by learning conditional probability of each attribute [35]. J48, also known as C4.5 grows an initial tree by using divide-and-conquer algorithm and then rank test instances [36]. Random Forest developed by Breiman [37] generates many classification trees simultaneously where each node uses a random subset of the features and outputs the classification based on majority voting over all trees in the forest. After comparison of these different classifiers by using Weka 3.6 [38], Random Forest algorithm was found to outperform other classifiers which were previously shown to be the best classification algorithm (e.g. SVM) [39].

Each classifier is trained by using a 10-fold cross-validation. Cross-validation measures the prediction performance in a stable way by leaving out a few instances (about 10% for 10-fold cross-validation) to be used as the test set during the training process. The exclusion is repeated until every instance in the dataset is once among those left-outs. In comparison to using an independent test set, cross-validation provides less bias and a better predictive performance. Parameter selection for each classifier is done by varying their parameters step-by-step and their accuracy and AUC (Area Under the ROC Curve) values are compared to obtain the best parameters with the highest performance (See additional file 2: Table S7 for parameter values used for classifier trainings). At the end, the classifier with the best performance is chosen as the model classifier.

The number of true positives (TP), true negatives (TN), false positives (FP) and false negatives (FN) are used to calculate true positive rate (also named as recall or sensitivity), TPR = TP/(TP + FN), false positive rate, FPR = FP/(FP + TN) and precision, P = TP/(TP + FP). We measure the performance of each classifier by using a ROC curve which is drawn as TPR (Sensitivity) versus FPR (1-Specificity). The area under the ROC curve, referred as AUC, represents the predictive power: while a random predictive model has an AUC = 0.5, a perfect one has an AUC = 1.0 so that a larger AUC shows a better predictive power. However, ROC curves can sometimes be misleading while dealing with highly unbalanced datasets. Therefore, Precision versus Recall (PR) curves are also constructed to interpret the performance of models in a more informative manner [40]. PR curves show how many true positives are likely to be obtained in a prediction system.

## Results

### Interaction prediction model

Random forest is chosen to build our model due to its highest AUC and accuracy values (See additional file 2: Figure S1 and Figure S2 for comparison of classifiers for trigram and bigram models, respectively). To optimize the parameters of Random Forest algorithm, we evaluate the effect of changes in parameters on its prediction performance by measuring out-of-bag (OOB) error rate of each model tree. There are two parameters: number of trees (numTree) and number of randomly selected features (numFeature). The number of features to be used in random selection is rather sensitive and it must be much lower than the total number of features [37]. On the other hand, the changes in the number of trees can result only in small decreases in OOB error rate. The lowest OOB error rate is obtained when numTree = 200 and numFeature = 30 (See additional file 2: Figure S5 for parameter selection graph). Also, resampling is applied as a pre-process in order to take care of our imbalanced dataset which could be overwhelmed by the major class otherwise and to derive robust estimates of standard errors. Resampling is a supervised filter producing a random subset of the dataset. In our study, class distribution is left as-is and sampling is done with replacement by adjusting the parameters.

**Figure 2:**
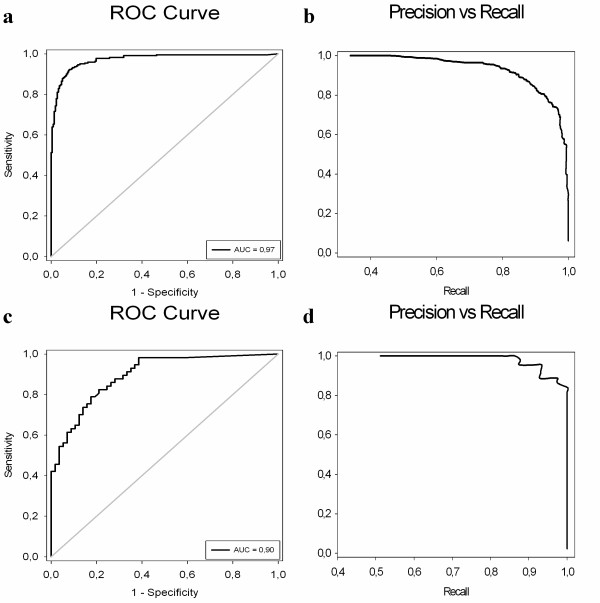
**Performance evaluation of Random Forest trigram model**. (a) ROC curve, (b) precision versus recall curve for interaction prediction part (c) ROC curve, (d) precision versus recall curve for classification part.

The accuracy of trigram part (91.4%) is slightly higher than the bigram part (91.2%) (Table 2). So, we design our model according to trigram frequency feature space. Our result of AUC = 0.97 for trigram part is high enough to be able to characterize PDZ binding specificity (Figure 2a). Also, we validate the power of our model by predicting the interaction of an unseen validation dataset. The model performs well on the validation set with an accuracy of 79.8% that it correctly classifies 25 of 27 binding and 46 of 62 non-binding interactions (Table 2). The performance of bigram model is somehow lower in validation dataset compared to trigrams. This may be due to the fact that bigrams assign more common features for most of the interactions by probably masking the discriminative features.

**Table 2:** Prediction results for interaction prediction of PDZ domains for both trigram and bigram models.

	Training set (10-fold cross validation)				Validation set			
		
	TPR	FPR	Precision	Accuracy	TPR	FPR	Precision	Accuracy
Trigram	0.89	0.075	0.85	91.4	0.61	0.042	0.92	79.8
Bigram	0.844	0.053	0.89	91.2	0.889	0.323	0.545	74.2

### Class prediction model

There is a multi-classification problem for class prediction because we do not only want to discriminate between PDZ domains which bind to Class I or Class II, but also we want to Class I-II domains whose interaction specificity reflects the promiscuous pattern of PDZ domains. All five classifiers are trained on these classification datasets and again Random Forest gives the best predictive performance with the highest AUC and accuracy values (See additional file 2: Figure S3 and Figure S4 for comparison of classifiers for trigram and bigram models, respectively).

The Random Forest model is used to discriminate both multi-classes (Class I/Class II/Class I-II) and binary classes (Class I/Class II, Class I/Class I-II or Class II/Class I-II) in order to have an insight about their pair wise classifications. As seen in Table 3, the predictive performance for multi-class learning is a bit lower than binary-class ones which are very close to each other and the trigram model has still better performance than those of the bigram model. As we are trying to distinguish all three classes of PDZ domains, we obtain the performance results of trigram model for multi-class learning (Figure 2c, 2d). The model correctly classifies 43 of 45 Class I, 16 of 20 Class II and 19 of 21 Class I-II PDZ domains. The results of binary comparisons, in Table 3, show that the highest accuracy is for differentiating Class II PDZs from Class I-II PDZs and the least successful one is between Class I and Class I-II. This means that amino acid distribution of Class I-II PDZs is slightly more similar to Class I PDZs. To ensure this similarity, amino acid frequency distribution histogram for Class I/II/I-II PDZ domains is plotted (See additional file 2: Figure S6 for amino acid frequency distribution).

**Table 3:** Prediction results for class prediction of PDZ domains for both trigram and bigram models.

	TP Rate		FP Rate		Precision		Accuracy (%)	
				
	Trigram	Bigram	Trigram	Bigram	Trigram	Bigram	Trigram	Bigram
ClassI, ClassII, Class I-II*	0.907	0.895	0.081	0.093	0.911	0.902	90.7	89.5
ClassI, ClassII	0.918	0.956	0	0.200	1	0.915	93.8	90.8
ClassI, ClassI-II	0.900	0.955	0	0.227	1	0.894	92.4	89.4
ClassII, ClassI-II	1	0.813	0.107	0	0.812	1	92.7	92.7

*The first row shows a multi-class learning and remaining rows shows the binary-class learning for pair wise combinations of three classes. For multi-class learning, weighted average results were shown.

### Dimensionality reduction

In order to make the resulting model faster and extract important features, dimensionality of our dataset is reduced by using feature selection methods [41]. Selection of important features can help us to get rid of redundant and/or irrelevant data. As the first step of feature selection, a correlation-based feature subset selection method is used to evaluate the individual performance of each feature for predicting labels (w_i_) as well as the level of intercorrelation among all features. Successful feature subsets include features highly correlated with the label, but uncorrelated with each other [42]. In the second step of feature selection part, several search algorithms are performed and results of all of them are considered in order to reduce features carefully. The search methods that are used are presented in additional file 2: Table S6.

Feature selection (dimension reduction) is applied to both trigram and bigram models because we want to observe important common features of both models. For the trigram model, we obtained 23 features for PDZ domain and 23 features for peptides to be used in interaction prediction part. Also, 53 trigram features, for the classification part, are obtained (Data is not shown).

For the bigram model, there are 11 features for PDZ domain and 12 features for peptides for the interaction prediction part and 10 features for the classification part. The accuracy values of our model did not increase after feature reduction for both interaction prediction and classification parts except trigram classification model (Table 4). However, reduction in feature space helps us analyze these selected important features to determine critical sequence motifs.

**Table 4:** Prediction results after feature reduction.

	TPR		FPR		Precision		AUC		Accuracy (%)	
					
	Trigram	Bigram	Trigram	Bigram	Trigram	Bigram	Trigram	Bigram	Trigram	Bigram
Interaction prediction	0.744	0.786	0.096	0.07	0.798	0.851	0.905	0.948	85	88.1
Classification*	0.942	0.86	0.044	0.096	0.942	0.859	0.994	0.966	94.2	86

* Weighted average result for multi-class learning (Class I, Class II, Class I-II)

### Critical sequence motifs

**Figure 3:**
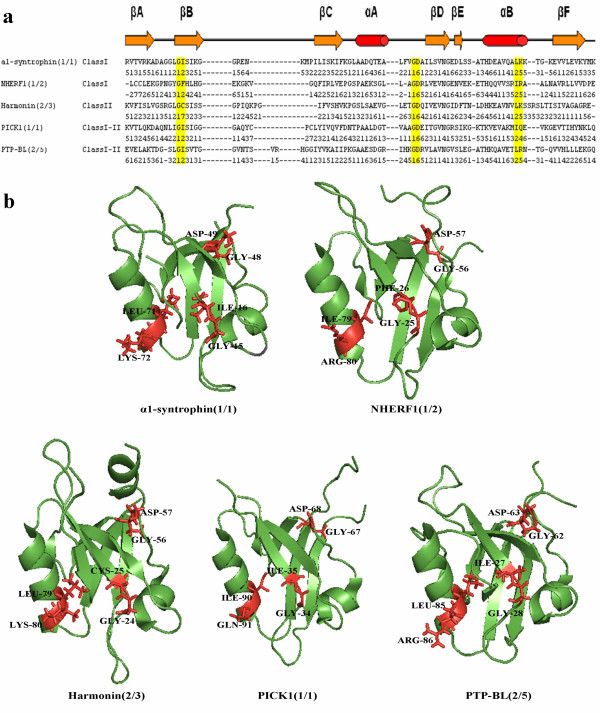
**Critical sequence motifs**. (a) Aligned sequences of 5 representative PDZ domains: α1-syntrophin(1/1) (PDB ID:2pdz), NHERF1(1/2) (PDB ID:1i92), Harmonin(2/3) (PDB ID:2kbs), Pick1(1/1) (PDB ID:2pku) and PTP-BL(2/5) (PDB ID:1vj6). While first row indicates the aligned sequence of corresponding PDZ domain, second row represents the sequence in seven class amino acid types. Secondary structure positions of the PDZ sequences are represented graphically at the top (αA, Αb, βA-βF). Three sequence motifs ("12", "16", "25") proposed to account for ligand specificity are indicated by yellow highlight. (b) Cartoon diagrams of these PDZ domains, motifs "12", "16" and "25" are colored in red and shown in stick form.

We analyzed the selected features to understand why they are important to distinguish different PDZ interactions and classes. These extracted features might correspond to some critical amino acid motifs which may be important for PDZ domain interaction specificity. Indeed, one of the features points out the GLGF (Gly-Leu-Gly-Phe) repeat of PDZ domains which is an important conserved region for peptide interaction [17]. This conserved GLGF sequence is located between βA-βB loop and αB helix and directly involved in peptide selectivity and binding. In order to determine important motifs, like the GLGF motif, we first find the common selected features of trigram and bigram models. Next, we perform multiple sequence alignment of all PDZ domains and we determine the conserved motifs (trigram, bigram features) on the similar secondary structure regions. We observe that conserved bigrams are also parts of the conserved trigrams, i.e. there are some highly occurring bigrams in both trigram and bigram feature sets for interaction prediction and classification parts. By looking at the most conserved ones, we obtain sequence motifs of "12", "16" and "25" (Figure 3) where the numbers in the motifs correspond to amino acid types and can be found in Table 1. For example, motif "12" corresponds to small hydrophobic amino acid (A, G, V) followed by large hydrophobic amino acid (I, L, F, P).

As seen in Figure 3, characteristic GLGF repeat of PDZ domains was determined by extracting sequence motif of "12" between βA-βB loop and αB helix. Other two highly occurring sequence motifs were positioned at the end of the αB ("25") and at the loop between αA and βD ("16"). When these sequence motifs are displayed on the 3D structure of PDZ domains, motif "25" is positioned near the binding groove (at the end of the αB), while motif "16" is positioned far from the binding groove (at the αA-βD loop) (Figure 3).

Extracted motif on αB helix could function in specificity of PDZ domains. Songyang *et al. *investigated the importance of αB helix on peptide selectivity of PDZ domains by showing high correlation between first residue in the αB helix and peptide position -2 [17]. Below, we discuss some specific PDZ domains:

#### α1-syntrophin(1/1)

The specific interaction property of α1-syntrophin PDZ domain was investigated by Schultz *et al. *and they found that Leu 14, Gly 15 and Ile 16 showed a large chemical shift upon binding of ligand [43]. PDZ domain of α1-syntrophin forms hydrophobic pocket consisting of Leu 14, Ile 18 and Leu 71 to bury the side chain of Val -2 of the peptide. Motif "12" corresponds to Gly 15, Ile 16 and "5" of motif "25" corresponds to Leu 71 which is an important part of the hydrophobic pocket.

#### NHERF(1/2)

First PDZ domain of NHERF1 plays important role in cellular localization by binding to the cystic fibrosis transmembrane conductance regulator (CFTR) [44]. Leu 0 of the ligand forms hydrophobic contact with Phe 26 and Ile 79 and makes H-bonds with Gly 25, Phe 26 and Arg 80. These important residues were also extracted by using our method: while motif "12" in βB corresponds to Gly 25, Phe 26, motif "25" in αB exactly corresponds to Ile 79, Arg 80.

#### Harmonin(2/3)

Pan *et al. *tried to elucidate structural basis of binding pattern of Harmonin(2/3) and found that carboxyl group of cad 23 ligand forms hydrogen bonds with Leu 222, Glu 223, Cys 224 (GLGF motif) and is stabilized by Lys 279 [45]. These important residues of Harmonin were also observed in our motifs as seen from Figure 3 (PDZ2 domain of Harmonin includes residues 208-299, but in the 3D structure it is between residues 9-100).

#### Pick1(1/1)

The carboxyl group of ligand forms hydrogen bonds with Ile 33, Gly 34 and Ile 35 of Pick1 PDZ domain [46]. While Gly 34 and Ile 35 constitute motif "12", we observed motif "24" on αB helix instead of motif "25".

#### PTP-BL(2/5)

Gianni *et al. *investigated allosteric property of PTP-BL(2/5) domain by using structural and dynamical methods and found that binding is regulated by long range interactions which showed correlation with ligand-induced structural rearrangements [47]. There is a detectable conformational change, dominantly occurring in αB-βB interface, L1 loop and hydrophobic core, upon ligand binding to PTP-BL domain. Plasticity and selectivity of PTP-BL domain are usually determined by reorientation of alpha B helix. Amides of Leu 25, Gly 26 and Ile 27 stabilize the charge of C-terminus of the ligand and there is a hydrophobic contact between C-terminal peptide valine and Leu 85, Val 82 positions. In our study, motif "12" in βB corresponds to the Gly 26, Ile 27 and "5" of motif "25" in αB corresponds to Leu 85 as seen Figure 3.

## Discussion

Our results show that our model can be used as a stable interaction prediction model of PDZ domains with higher accuracy than other similar methods [22,24]. We also proposed a classification model for PDZ domains based on the general classification pattern unlike other methods [15,25] and the result with high accuracy indicates that our classification model highly correlates with the current classification pattern of PDZ domains. Although PDZ domains show highly selective interaction pattern, there are some PDZ domains which bind to both Class I and Class II peptides. We named these promiscuous PDZ domains as Class I-II PDZ domains and obtained a very high performance when discriminating them among other classes. Therefore, it is concluded that there may be some characteristic pattern in the structure of Class I-II PDZ domains that provide its promiscuous property.

Some important characteristic features of PDZ domains were extracted. After selection of most occurring features along the same secondary structure region of PDZ domains, we obtained three critical sequence motifs. Two of them ("12" and "25") were previously shown to have an important role in ligand interaction. Motif "12" is on conserved GLGF repeat and located between βA-βB loop and motif "25" is located on αB which is one of the parts of binding pocket. There is not any previous study investigating the importance of motif "16" which is positioned on αA-βD loop. After multiple alignment of PDZ domain sequences, it was observed that motif "16" on αA-βD loop is conserved as shown in another study [48]. Although this motif does not locate near the canonical binding pocket, it could be involved in dimerization of PDZ domains which is a common characteristic for some PDZ domains [48-51]. In the study of Im *et al.*, it was shown that the dimeric interface of GRIP1 PDZ6 dimer include a βA strand and αA-βD loop from each domain, and motif "16" is located on this αA-βD loop of GRIP1 PDZ6 domain. Also, it could have an allosteric effect regulating the binding specificity of PDZ domains [52]. However, further study has to be performed in order to reveal biological importance of this motif.

## Conclusions

This study has two intercorrelated aims: prediction of PDZ domain-peptide interactions, and classification of PDZ domains as Class I, II and I-II. A statistical learning model was constructed by using interaction dataset of PDZ domains (consist of 85 PDZ domains and corresponding 181 peptides). To convert primary sequence information into numerical feature input, trigram and bigram amino acid frequencies were calculated for each instance. We predicted binary interactions and classes of PDZ domains with accuracies of 91.4% and 90.7%, respectively. After feature extraction, three critical amino acid sequence motifs were proposed to have significant roles on PDZ domain specificity. With these highly encouraging results, this study could be an important step in the automated prediction of PDZ domain interactions.

The discovery of features within primary sequences of known protein interaction pairs could be subsequently developed by using other features (binding affinities, secondary/tertiary structure, etc.) in the learning model. Further improvements on these lines may generate a powerful computational virtual screening technique that significantly reduces the search space for putative candidate target proteins of PDZ domains.

## Authors' contributions

SK, OK and AG designed the study. SK implemented the algorithms, performed the experiments. SK, OK and AG analyzed the data. All authors have read and approved the final manuscript.

## Supplementary Material

Additional file 1 **Table S1 - PDZ domain interactions**. Click here for file

Additional file 2 **Table S2 - Unseen validation dataset interactions**. Table S3 - Classes of PDZ domains according to their binding/non-binding target peptide sequences. Table S4 - PDZ domain sequence IDs. Table S5 - Peptide sequences (up to -10 amino acid position). Table S6 - Search methods that are used to reduce dimensionality. Table S7 - Parameter values for each classifier used in trigram interaction prediction model. Figure S1 - Comparison of all classifiers used in interaction prediction trigram model. Figure S2 - Comparison of all classifiers used in interaction prediction bigram model. Figure S3 - Comparison of all classifiers used in classification trigram model. Figure S4 - Comparison of all classifiers used in classification bigram model. Figure S5 - Parameter selection of Random Forest algorithm for interaction prediction trigram model. Figure S6. Amino acid frequency distribution of Class I/II/I-II PDZ domains. Click here for file
