# Supplementary material for: Interaction prediction and classification of PDZ domains
Source: BMC Bioinformatics. 2010 Jun 30;11:357. doi: 10.1186/1471-2105-11-357 (PMC2909223; doi:10.1186/1471-2105-11-357)
Supplement: Additional file 2 — Table S2 - Unseen validation dataset interactions. Table S3 - Classes of PDZ domains according to their binding/non-binding target peptide sequences. Table S4 - PDZ domain sequence IDs. Table S5 - Peptide sequences (up to -10 amino acid position). Table S6 - Search methods that are used to reduce dimensionality. Table S7 - Parameter values for each classifier used in trigram interaction prediction model. Figure S1 - Comparison of all classifiers used in interaction prediction trigram model. Figure S2 - Comparison of all classifiers used in interaction prediction bigram model. Figure S3 - Comparison of all classifiers used in classification trigram model. Figure S4 - Comparison of all classifiers used in classification bigram model. Figure S5 - Parameter selection of Random Forest algorithm for interaction prediction trigram model. Figure S6. Amino acid frequency distribution of Class I/II/I-II PDZ domains. [file 1471-2105-11-357-S2.PDF]

ADDITIONAL FILE 2

Table S2. Unseen validation dataset interactions. 1
Table S3. Classes of PDZ domains according to their binding/non-binding target peptide sequences 2
Table S4. PDZ domain sequence IDs 3
Table S5. Peptide sequences (up to -10 amino acid position). 4
Table S6. Search methods that are used to reduce dimensionality 6
Table S7. Parameter values for each classifier used in trigram interaction prediction model. 6

Figure S1. Comparison of all classifiers used in interaction prediction trigram model (a) Accuracy values with 95% confidence intervals (b) ROC curves and corresponding AUC values. 7
Figure S2. Comparison of all classifiers used in interaction prediction bigram model (a) Accuracy values with 95% confidence intervals (b) ROC curves and corresponding AUC values. 7
Figure S3. Comparison of all classifiers used in classification trigram model (the result of multi-classification to discriminate between Class I, Class II and Class I-II). (a) Accuracy values with 95% confidence intervals (b) ROC curves and corresponding AUC values. 8
Figure S4. Comparison of all classifiers used in classification bigram model (the result of multi-classification to discriminate between Class I, Class II and Class I-II). (a) Accuracy values with 95% confidence intervals (b) ROC curves and corresponding AUC values. 8
Figure S5. Parameter selection of Random Forest algorithm for interaction prediction trigram model. 9
Figure S6. Amino acid frequency distribution of Class I/II/I-II PDZ domains 9

Table S2. Unseen validation dataset interactions.

PDZ domain	Peptide	Interaction
PSD95-(1/3)	Scn4a	binding
PSD95-(2/3)	Scn4a	binding
PSD95-(3/3)	Scn4a	binding
SAP97-(1/3)	Scn4a	binding
SAP97-(2/3)	Scn4a	binding
SAP97-(3/3)	Scn4a	binding
Chapsyn-110-(2/3)	Scn4a	binding
Chapsyn-110-(3/3)	Scn4a	binding
SAP102-(2/3)	Scn4a	binding
CIPP-(2/10)	Scn4a	binding
nNOS	Scn4a	binding
α1-syntrophin	Scn4a	binding
PSD95-(2/3)	Mapk12	binding
PSD95-(3/3)	Mapk12	binding
SAP97-(2/3)	Mapk12	binding
SAP97-(3/3)	Mapk12	binding
Chapsyn-110-(2/3)	Mapk12	binding
Chapsyn-110-(3/3)	Mapk12	binding
SAP102-(2/3)	Mapk12	binding
α1-syntrophin	Mapk12	binding
PSD95-(1/3)	Scn5a	binding
PSD95-(2/3)	Scn5a	binding
SAP97-(2/3)	Scn5a	binding
Chapsyn-110-(2/3)	Scn5a	binding
SAP102-(2/3)	Scn5a	binding
nNOS	Scn5a	binding
α1-syntrophin	Scn5a	binding
Chapsyn-110-(1/3)	Cript	non-binding
Chapsyn-110-(1/3)	Dlgap1/2/3	non-binding
Chapsyn-110-(1/3)	Stargazin	non-binding
Chapsyn-110-(1/3)	Scn5a	non-binding
Chapsyn-110-(1/3)	L-glutaminase	non-binding
Chapsyn-110-(1/3)	AN2	non-binding
Chapsyn-110-(1/3)	Parkin	non-binding
Chapsyn-110-(1/3)	EphrinB1/2	non-binding
Chapsyn-110-(1/3)	Nrxn1/2	non-binding
Chapsyn-110-(1/3)	GluR2	non-binding
Chapsyn-110-(1/3)	Claudin1	non-binding
Chapsyn-110-(1/3)	Mel1a/b	non-binding
CIPP-(2/10)	NMDAR2A	non-binding
CIPP-(2/10)	Dlgap1/2/3	non-binding
CIPP-(2/10)	Kv1.4	non-binding
CIPP-(2/10)	Frizzled	non-binding
CIPP-(2/10)	Cnksr2	non-binding
CIPP-(2/10)	KIF1B	non-binding
CIPP-(2/10)	Mapk12	non-binding
CIPP-(2/10)	Scn5a	non-binding
CIPP-(2/10)	L-glutaminase	non-binding
CIPP-(2/10)	Kir2.1	non-binding
CIPP-(2/10)	AN2	non-binding
CIPP-(2/10)	Parkin	non-binding
CIPP-(2/10)	EphrinB1/2	non-binding
CIPP-(2/10)	Nrxn1/2	non-binding
CIPP-(2/10)	GluR2	non-binding
CIPP-(2/10)	Claudin1	non-binding
CIPP-(2/10)	Mel1a/b	non-binding
CASK	Cript	non-binding
CASK	Dlgap1/2/3	non-binding
CASK	Scn4a	non-binding
CASK	Stargazin	non-binding
CASK	Frizzled	non-binding
CASK	Cnksr2	non-binding
CASK	KIF1B	non-binding
CASK	Mapk12	non-binding
CASK	Scn5a	non-binding

CASK	Kir2.1	non-binding
CASK	AN2	non-binding
CASK	EphrinB1/2	non-binding
CASK	GluR2	non-binding
CASK	Claudin1	non-binding
CASK	Mel1a/b	non-binding
GRIP1-(7/7)	NMDAR2A	non-binding
GRIP1-(7/7)	Cript	non-binding
GRIP1-(7/7)	Dlgap1/2/3	non-binding
GRIP1-(7/7)	Scn4a	non-binding
GRIP1-(7/7)	Kv1.4	non-binding
GRIP1-(7/7)	Stargazin	non-binding
GRIP1-(7/7)	Frizzled	non-binding
GRIP1-(7/7)	Cnksr2	non-binding
GRIP1-(7/7)	KIF1B	non-binding
GRIP1-(7/7)	Mapk12	non-binding
GRIP1-(7/7)	Scn5a	non-binding
GRIP1-(7/7)	L-glutaminase	non-binding
GRIP1-(7/7)	Kir2.1	non-binding
GRIP1-(7/7)	Parkin	non-binding
GRIP1-(7/7)	EphrinB1/2	non-binding
GRIP1-(7/7)	GluR2	non-binding
GRIP1-(7/7)	Claudin1	non-binding
GRIP1-(7/7)	Mel1a/b	non-binding

Table S3. Classes of PDZ domains according to their binding/non-binding target peptide sequences.

PDZ domain	Organism	Class
a1-syntrophin-(1/1)	mouse	ClassI
b1-syntrophin-(1/1)	mouse	ClassI
Chapsyn-110-(2/3)	mouse	ClassI
Chapsyn-110-(3/3)	mouse	ClassI
Erbin-(1/1)	mouse	ClassI
g1-syntrophin-(1/1)	mouse	ClassI
g2-syntrophin-(1/1)	mouse	ClassI
Interleukin-16-(1/4)	mouse	ClassI
LIN-7A-(1/1)	mouse	ClassI
Lin7c-(1/1)	mouse	ClassI
Lrrc7-(1/1)	mouse	ClassI
Magi-1-(2/6)	mouse	ClassI
Magi-1-(4/6)	mouse	ClassI
Magi-2-(2/6)	mouse	ClassI
Magi-2-(5/6)	mouse	ClassI
Magi-3-(2/5)	mouse	ClassI
Mals2-(1/1)	mouse	ClassI
MUPP1-(12/13)	mouse	ClassI
MUPP1-(13/13)	mouse	ClassI
NHERF-1-(1/2)	mouse	ClassI
NHERF-2-(2/2)	mouse	ClassI
nNOS-(1/1)	mouse	ClassI
OMP25-(1/1)	mouse	ClassI
PAR6B-(1/1)	mouse	ClassI
Pdlim5-(1/1)	mouse	ClassI
Pdzk1-(1/4)	mouse	ClassI
Pdzk1-(3/4)	mouse	ClassI
Pdzk11-(1/1)	mouse	ClassI
PSD95-(1/3)	mouse	ClassI
PSD95-(2/3)	mouse	ClassI
PSD95-(3/3)	mouse	ClassI
SAP102-(2/3)	mouse	ClassI
SAP102-(3/3)	mouse	ClassI
SAP97-(1/3)	mouse	ClassI
SAP97-(2/3)	mouse	ClassI
SAP97-(3/3)	mouse	ClassI
Scrb1-(1/4)	mouse	ClassI
Scrb1-(2/4)	mouse	ClassI
Scrb1-(3/4)	mouse	ClassI
Semcap3-(1/2)	mouse	ClassI
Shank3-(1/1)	mouse	ClassI
Shroom-(1/1)	mouse	ClassI
SLIM-(1/1)	mouse	ClassI
TIP-1-(1/1)	mouse	ClassI
Whirlin-(3/3)	mouse	ClassI
ABP-(3/7)	rat	ClassII
ABP-(5/7)	rat	ClassII
ABP-(6/7)	rat	ClassII
AF-6-(1/1)	human	ClassII

ASIP/PAR3-(1/3)	mouse	ClassII
CASK-(1/1)	human	ClassII
ZO-1-(2/3)	human	ClassII
p55-(1/1)	mouse	ClassII
Cipp-(5/10)	mouse	ClassII
Cipp-(9/10)	mouse	ClassII
D930005D10Rik-(1/1)	mouse	ClassII
Dlgh3-(1/1)	mouse	ClassII
Grip1-(6/7)	mouse	ClassII
Harmonin-(2/3)	mouse	ClassII
Mpp7-(1/1)	mouse	ClassII
MUPP1-(1/13)	mouse	ClassII
MUPP1-(5/13)	mouse	ClassII
MUPP1-(11/13)	mouse	ClassII
PAR-3-(3/3)	mouse	ClassII
ZO-1-(2/3)	mouse	ClassII
Cipp-(3/10)	mouse	ClassI-II
Cipp-(8/10)	mouse	ClassI-II
Cipp-(10/10)	mouse	ClassI-II
Dvl1-(1/1)	mouse	ClassI-II
Dvl2-(1/1)	mouse	ClassI-II
Dvl3-(1/1)	mouse	ClassI-II
GRASP55-(1/1)	mouse	ClassI-II
HtrA1-(1/1)	mouse	ClassI-II
HtrA3-(1/1)	mouse	ClassI-II
Lnx1-(2/4)	mouse	ClassI-II
Magi-1-(6/6)	mouse	ClassI-II
Magi-2-(6/6)	mouse	ClassI-II
Magi-3-(1/5)	mouse	ClassI-II
Magi-3-(5/5)	mouse	ClassI-II
MUPP1-(10/13)	mouse	ClassI-II
PDZ-RGS3-(1/1)-	mouse	ClassI-II
PTP-BL-(2/5)	mouse	ClassI-II
ZO-1-(1/3)	mouse	ClassI-II
ZO-2-(1/3)	mouse	ClassI-II
PICK1-(1/1)	human	ClassI-II
Syntenin-(2/2)	mouse	ClassI-II

Table S4. PDZ domain sequence IDs

PDZ domain	Sequence ID
a1-syntrophin-(1/1)	Q61234
b1-syntrophin-(1/1)	Q99L88
Chapsyn-110-(2/3)	Q91XM9
Chapsyn-110-(3/3)	Q91XM9
Cipp-(3/10)	Q63ZW7
Cipp-(5/10)	Q63ZW7
Cipp-(8/10)	Q63ZW7
Cipp-(9/10)	Q63ZW7
Cipp-(10/10)	Q63ZW7
D930005D10Rik-(1/1)	Q69Z89
Dlgh3-(1/1)	O88910
Dvl1-(1/1)	P51141
Dvl2-(1/1)	Q60838
Dvl3-(1/1)	Q61062
Erbin-(1/1)	Q80TH2
g1-syntrophin-(1/1)	Q925E1
g2-syntrophin-(1/1)	Q925E0
Gm1582-(2/3)	UPI0000D670BC (196-264)
GRASP55-(1/1)	Q99JX3
Grip1-(6/7)	Q925T6
Grip2-(5/7)	UPI00001E3EA7 (431-504)
Harmonin-(2/3)	Q9ES64
HtrA1-(1/1)	Q9R118
HtrA3-(1/1)	Q9D236
Interleukin-16-(1/4)	Q9QZP6
LARG-(1/1)	UPI0000D63612 (296-364)
LIN-7A-(1/1)	Q8JZS0
Lin7c-(1/1)	O88952
Lnx1-(2/4)	O70263
Lrrc7-(1/1)	Q80TE7
Magi-1-(2/6)	Q6RHR9
Magi-1-(4/6)	Q6RHR9
Magi-1-(6/6)	Q6RHR9
Magi-2-(2/6)	Q9WVQ1
Magi-2-(5/6)	Q9WVQ1
Magi-2-(6/6)	Q9WVQ1
Magi-3-(1/5)	Q9EQJ9
Magi-3-(2/5)	Q9EQJ9
Magi-3-(5/5)	Q9EQJ9
Mals2-(1/1)	O88951
Mpp7-(1/1)	Q8BVD5
MUPP1-(1/13)	Q8VBX6
MUPP1-(5/13)	Q8VBX6
MUPP1-(10/13)	Q8VBX6
MUPP1-(11/13)	Q8VBX6
MUPP1-(12/13)	Q8VBX6
MUPP1-(13/13)	Q8VBX6
NHERF-1-(1/2)	P70441
NHERF-2-(2/2)	Q9JHL1
nNOS-(1/1)	Q9Z0J4

OMP25-(1/1)	Q8K4F3
PAR-3-(3/3)	Q99NH2
PAR3B-(1/3)	Q8TEW8
PAR6B-(1/1)	Q9JK83
Pdlim5-(1/1)	Q8CI51
Pdzk1-(1/4)	Q9JIL4
Pdzk1-(3/4)	Q9JIL4
Pdzk11-(1/1)	Q9CZG9
Pdzk3-(1/1)	ENSMUSP00000074788
Pdzk3-(2/2)	ENSMUSP00000043100
PDZ-RGS3-(1/1)	P49796
PSD95-(1/3)	Q62108
PSD95-(2/3)	Q62108
PSD95-(3/3)	Q62108
PTP-BL-(2/5)	Q64512
SAP102-(2/3)	P70175
SAP102-(3/3)	P70175
SAP97-(1/3)	Q811D0
SAP97-(2/3)	Q811D0
SAP97-(3/3)	Q811D0
Scrb1-(1/4)	Q80U72
Scrb1-(2/4)	Q80U72
Scrb1-(3/4)	Q80U72
Semcap3-(1/2)	Q69ZS0
Shank1-(1/1)	Q9Y566
Shank3-(1/1)	Q4ACU6
Shroom-(1/1)	Q9QXN0
SLIM-(1/1)	Q8R1G6
Tiam2-(1/1)	ENSMUSP00000024562
TIP-1-(1/1)	Q9DBG9
Whirlin-(3/3)	Q80VW5
ZO-1-(1/3)	P39447
ZO-1-(2/3)	P39447
ZO-2-(1/3)	Q9Z0U1
ZO-3-(1/3)	UPI00005652A2
ABP-(3/7)	Q9WTW1
ABP-(5/7)	Q9WTW1
ABP-(6/7)	Q9WTW1
AF-6-(1/1)	P55196
ASIP/PAR3-(1/3)	Q99NH2
CASK-(1/1)	O14936
ZO-1-(2/3)	Q07157
p55-(1/1)	P70290
PICK1-(1/1)	Q9NRD5
Syntenin-(2/2)	O08992

Table S5. Peptide sequences (up to -10 amino acid position).

Peptide	Sequence
AcvR1	NSLDKLKTDC
AcvR2	VDFPPKESSL
AcvR2b	VDLLPKESSI
AN2	PALRNGQYWV
APC	HSGSYLVTSV
Aquaporin-4	DSSGEVLSSV
AXL	PAPPGQEDGA
Cacna1a	AYSESEDDWC
Caspr2	IDESKKEWLI
Caspr4	VGENQKEYFF
Cav1.2	ADSRSYVSNL
Cav2.2	YHHPDQDHWC
Cav2.3	LSDTEEDDKC
Cav3.2	APDDSGDEPV
Cftr	TEEEVQETRL
c-KIT	TQPLLVHEDA
Claudin-1	PTPSSGKDYV
Claudin-10	SKQFDKNAYV
Claudin-11	SPTHAKSAHV
Claudin-14	HSGYRLNDYV
Claudin-15	FGKYGKNAYV
Claudin-16	AKMYAVDTRV
Claudin-18	QSHPTKYDYV
Claudin-19	GPSTAAREYV
Claudin-2	FNSYSLTGYV
Claudin-22	LELKQANPEI
Claudin-23	QNSLPCDSDL
Claudin-3	GTAYDRKDYV
Claudin-4	ARSVPASNYV
Claudin-5	NGDYDKKNYV
Claudin-6	PSEYPTKNYV
Claudin-7	PKSNSSKEYV
Claudin-8	PSIYSKSQYV
Claudin-9	ASGLDKRDYV
CNGA2	INTPEPAVAE
CNGA3	ENSEDASKTD
Cnksr2	HTHSYIETHV
Connexin-43	SRPRPDDLEI
CRIPT	DTKNYKQTSV
CSF-1R	LLQPNNYQFC
CtBP1	ADRDHTSDQL
DDR1	FLADDALNTV
Dlgap1/2/3	IYIPEAQTRL
EGFR	APPSSEFIGA
EphA2	DQVNTVGIPI

EphA3	TQSKNGPVPV
EphA4	QQMHGRMVPV
EphA5	VQMVNGMVPV
EphA6	MHIQEKGFHV
EphA7_1	LHLHGTGIQV
EphA7_2	LVTNEHLSVL
EphB2	QMNQIQSVEV
EphB3	QMNQTLPVQV
EphB6_1	HLRQPGSVEV
Ephrin-B1/2	QSPANIYYKV
Ephrin-B3	QSPPNIYYKV
ErbB4	VAQGATAEMF
FGFR3	GPPSNGGPRT
FGFR4	PFPFSDSQTT
Frizzled	TNSKQGETTV
GluR1	SGMPLGATGL
GluR2_1	NVYGIESVKI
GluR2_2	GMNVSVTDLS
GluR2_3	PKGTSLGWVE
GluR3	NVYGTESVKI
GluR5_1	RRTQRKETVA
GluR5_2	IRTQPSVHTV
GluRdelta1	ALDTSHGTSI
GluRdelta2	GNDPDRGTSI
Glycophorin-C	GDTSKKEYFI
GRK6	DSEEELPTRL
Htr2c	NVVSERISSV
JAM-1	EFKQTSSFLV
KA-2	TGPRELTEHE
KCNAB2	KPYSKKDYRS
KCNE4_1	RQAEGLVSIC
KCNE4_2	GSSENIHQNS
KCNH1	ESDRDIFGAS
KCNK3	RGLMKRRSSV
KCNK4_1	LEDFIKAMAI
KCNK4_2	GRLRDKAVPV
KCNK5	YNKADNPRGT
KCNK6	GPEREAPRSA
KCNQ2	PGTPRVTSQL
KIF17	SKNSFGGEPL
KIF1B	NLKAGRETTV
Kir2.1	PRPLRRESEI
Kir2.2	VRPYRRESEI
Kir3.2_2	VANLENESKV
Kir3.2_3	NPEELTERNG
Kir3.3	LPPPESESKV
Kir4.1	SALSVRISNV
Kir4.2	RSLLLQQSNV
Kir5.1	LNRISMESQM
Kir6.1	PEGNQCPSES
Kir6.2	KFSISPDSLS

Kv1.1	VNKSKLLTDV
Kv1.2	VNITKMLTDV
Kv1.3	VNIKKIFTDV
Kv1.4	SNAKAVETDV
Kv1.5	CLDTSRETDL
Kv1.6	YAEKRMLTEV
Kv1.7	PAGKHMVTEV
Kv2.1	AHGSTRDQSI
Kv3.1	GRKPLRGMSI
Kv3.3_1	RAPPTLPSIL
Kv3.3_2	FGERDSETQV
Kv4.1	LPETVKISSL
Kv4.2	GGNIVRVSAL
L-glutaminase	LSKENLESMV
Liprin-a2	DNSTVRTYSC
Megalin	ANLVKEDSDV
Mel1a/b	NNNLIKVDSV
mGluR1	RDYKQSSSTL
mGluR3	EVLDSTTSSL
Na/Pi-cotransporter	LPAHHNATRL
Nav1.4	VRPGVKESLV
Nav1.5	SPDRDRESIV
Nav1.6	RQKEVRESKC
Nav2	EEKASIQTQI
Neurexin-1/2	KKNKDKEYYV
Neurexin-3	QKNKDKEYYV
Neurexin-4	PQILEESRSE
Neuroligin-2	LPHPHSTTRV
NHE1	EGEPFIPKGQ
NMDAR2A	KKMPSIESDV
NMDAR2B	EKLSSIESDV
NMDAR2C	RRISSLESEV
NMDAR2D	AHFSSLESEV
P2Y1	EFKQNGDTSL
Parkin	ACMGDHWFDV
PDGFR	PLAEAEDSFL
PDGFRa_1	SSDLVEDSFL
PDGFRa_2	HSGKYDLSVV
PFK-M	SRKRSGEAAV
PIX	NDPAWDETNL
PKC	FVHPILQSAV
PMCA1	SPLHSLETSL
PTK7	LGDSPADSKQ
Ril	VYPNAKVELV
ROR1	HTESMISAEV
ROR2	TEAAHVQLEA
RYK	EFHAALGAYV
Sapk3	GARVPKETAL
Sema3a	HEFERAPRSV
Sema3b	ERGPRSAAHW

Sema3f	RNRRHHPPDT
Sema4a-	DNNHLGAEVA
Sema4b-	LGSEIRDSVV
Sema4c	PDSNPEESSV
Sema4f	PLATCDETSI
Sema5a	FTDLNNYDEY
Sema6b-	TGERTAPPVP
Sema6c	PAPHGGHFNF
SERCA2A	NYLEQPAILE
SERCA3	RGESPVWPSD
SSTR2	SGAEDIIAWV
Stargazin	NTANRRTTPV
Syndecan-1	KPTKQEEFYA
Syndecan-2	QKAPTKEFYA
Syndecan-3	KPDKQEEFYA
TAZ	NKSEPFLTWL
TIE1	AGIDATAEEA
TPC1	GSRQRSQTVT
Trip6	ELSATVTTDC

TRPC1	SKYAMFYPRN
TRPC2	EGDLETKGES
TRPC3	KLNPSVLRCE
TRPC4	AHEDYVTTRL
TRPC5	GQEEQVTTRL
TRPC6	LEPKLEESRR
TRPM3	DPAEHPFYSV
TRPM5	SQPLLETGST
TRPM6	RSSLEDHTRL
TRPM7	EATNSVRLML
TRPM8	LLKEIANNIK
TRPP2	SGNGSANVHA
TRPV3	ELDEFPETSV
TRPV4	PKWRTDDAPL
TRPV6	EDGEGWEYQI
TYRO3	QQGLLPHSSC

Table S6. Search methods that are used to reduce dimensionality.

Search Method	Description
**Best First**	Searches the space of attribute subsets by greedy hillclimbing augmented with a backtracking facility
**Greedy Stepwise**	Performs a greedy forward or backward search through the space of attribute subsets
**Linear Forward Selection**	Extension of BestFirst. Takes a restricted number of k attributes into account. Fixed-set selects a fixed number k of attributes, whereas k is increased in each step when fixed-width is selected
**Rank Search**	From the ranked list of attributes, subsets of increasing size are evaluated, ie. The best attribute, the best attribute plus the next best attribute, etc.
**Subset Size Forward Selection**	The search performs an interior cross-validation (here 5-fold is used). A Linear Forward Selection is performed on each fold to determine the optimal subset-size

Table S7. Parameter values for each classifier used in trigram interaction prediction model.

Classifier	Parameters
**SVM**	Complexity parameter: 1.0<br>Tolerance parameter: 0.001<br>Epsilon: 10^{-12}<br>Kernel: Linear kernel (Exponent: 1.0)
**Nearest Neighbor**	k (number of neighbors to use): 1<br>Distance function: Eucledian distance
**Naïve Bayes**	Alpha: 0.5 (Simple Estimator)
**J48**	Confidence factor: 0.25<br>Min number of objects per leaf: 2
**Random Forest**	Number of trees: 200<br>Number of Features:30

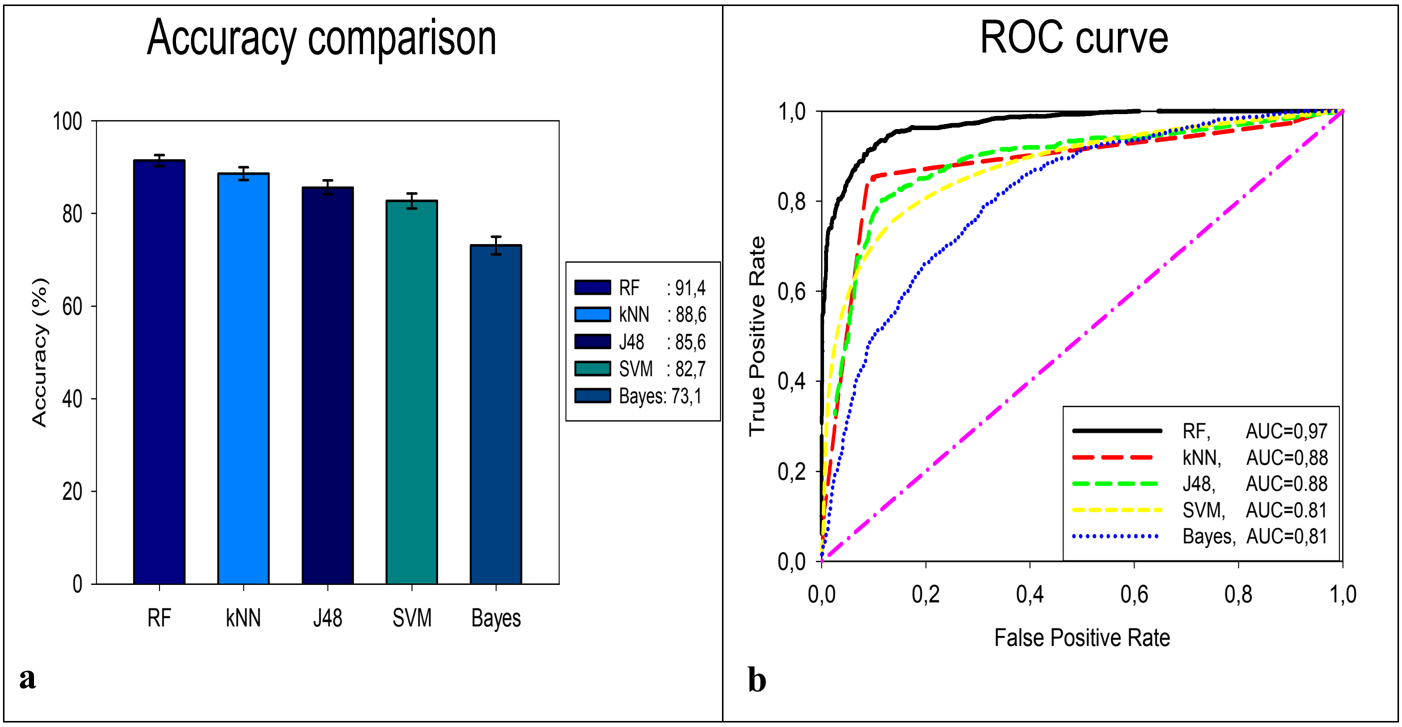

Figure S1. Comparison of all classifiers used in interaction prediction trigram model (a) Accuracy values with 95% confidence intervals (b) ROC curves and corresponding AUC values.

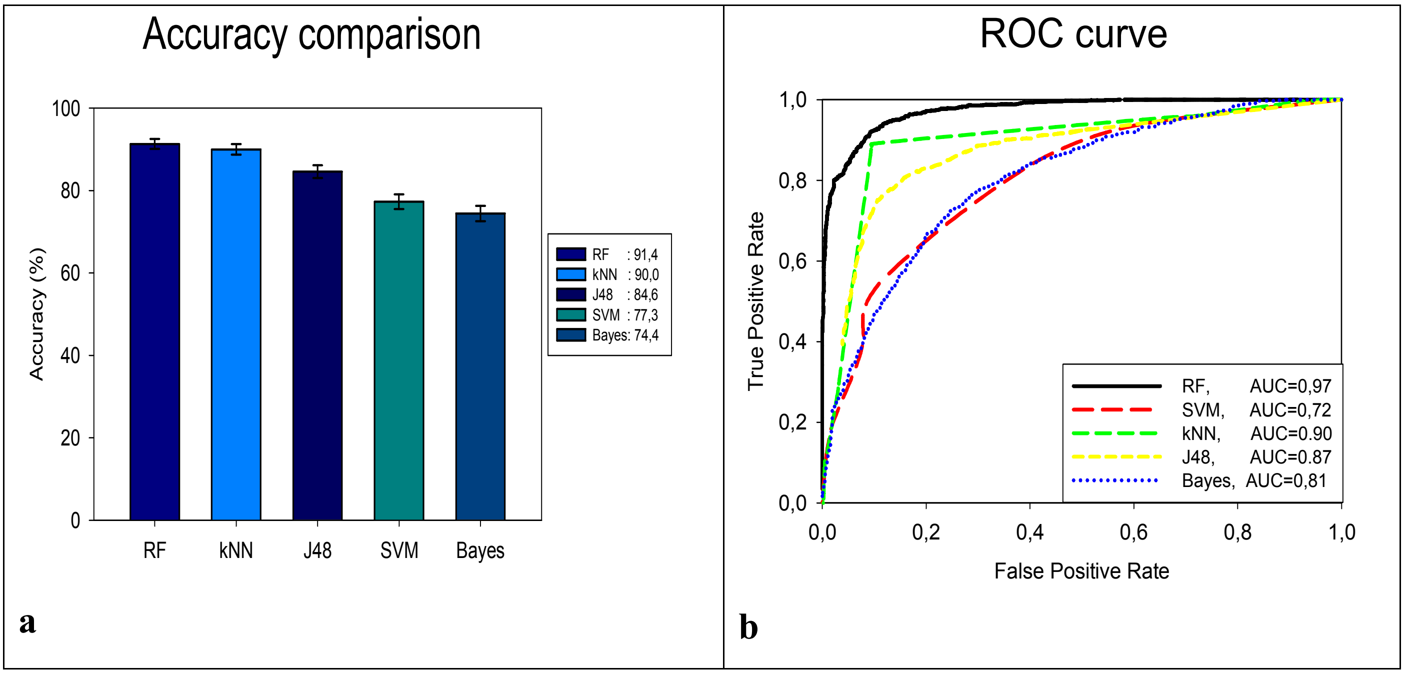

Figure S2. Comparison of all classifiers used in interaction prediction bigram model (a) Accuracy values with 95% confidence intervals (b) ROC curves and corresponding AUC values.

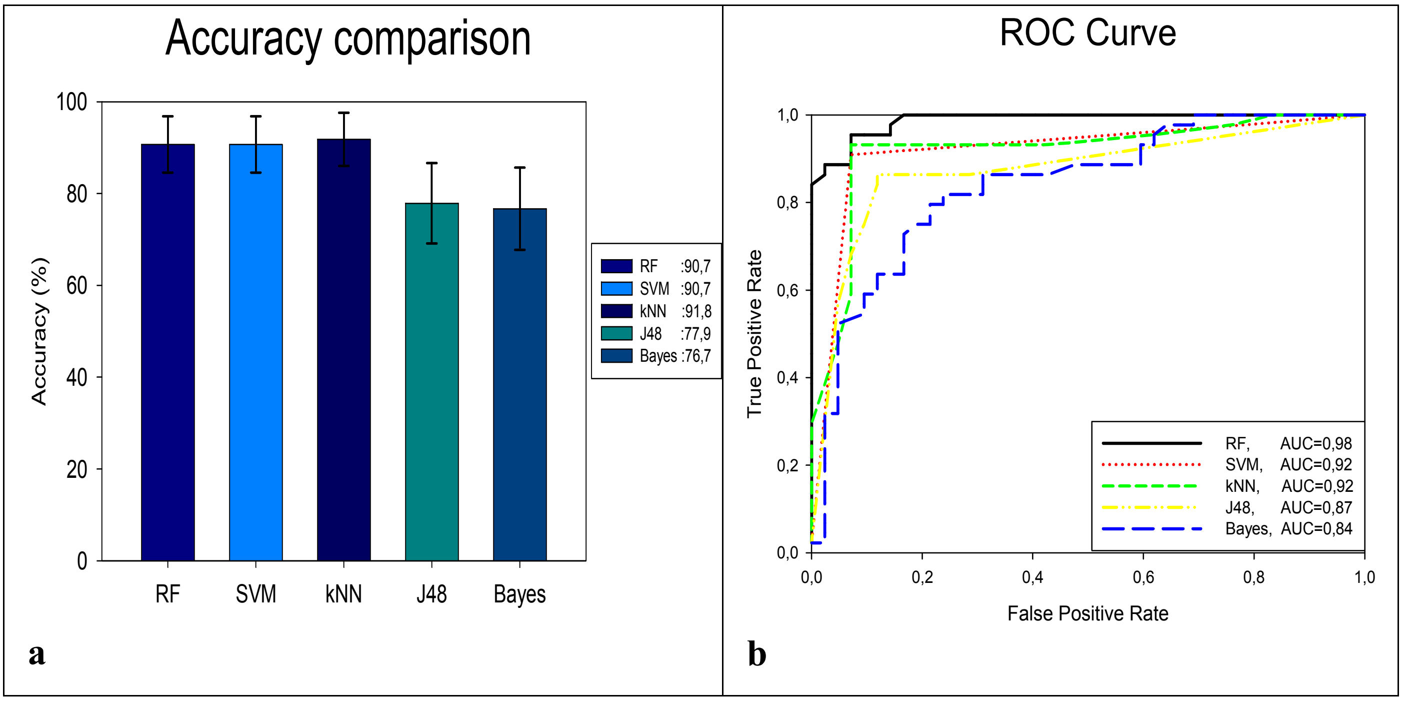

Figure S3. Comparison of all classifiers used in classification trigram model (the result of multi-classification to discriminate between Class I, Class II and Class I-II). (a) Accuracy values with 95% confidence intervals (b) ROC curves and corresponding AUC values.

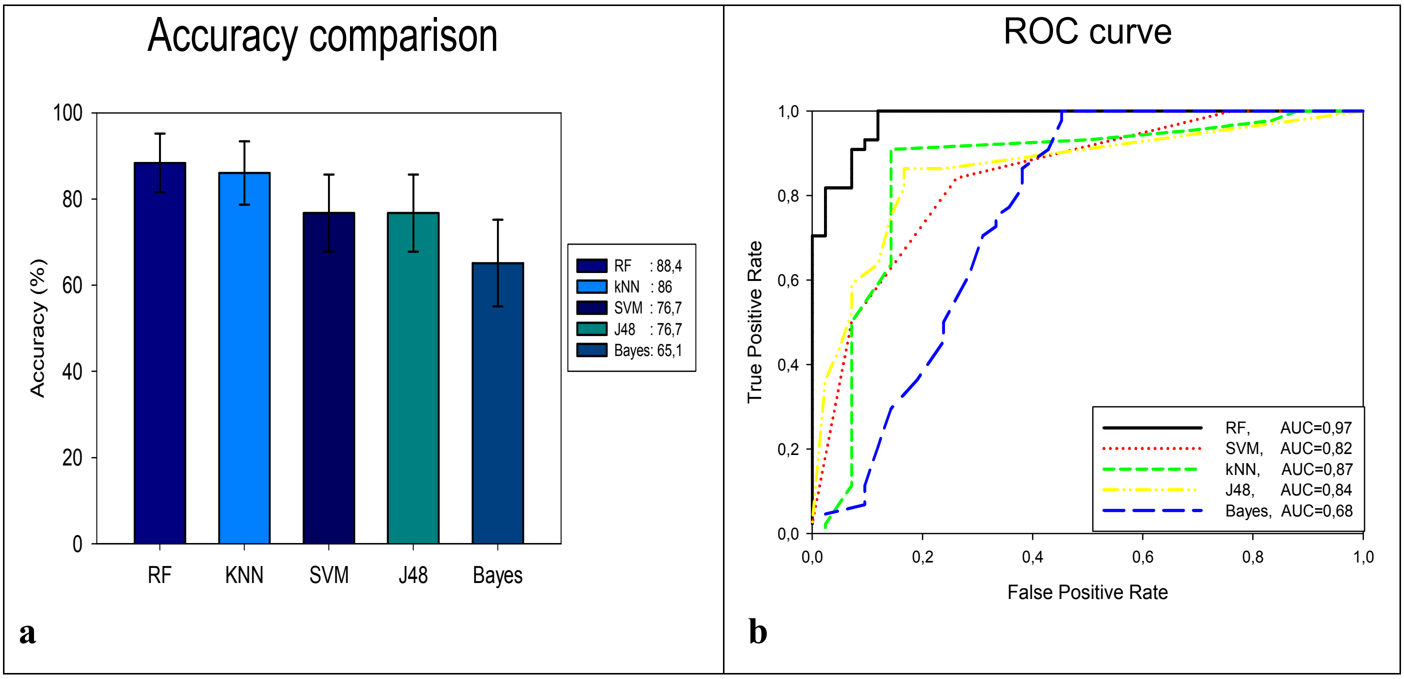

Figure S4. Comparison of all classifiers used in classification bigram model (the result of multi-classification to discriminate between Class I, Class II and Class I-II). (a) Accuracy values with 95% confidence intervals (b) ROC curves and corresponding AUC values.

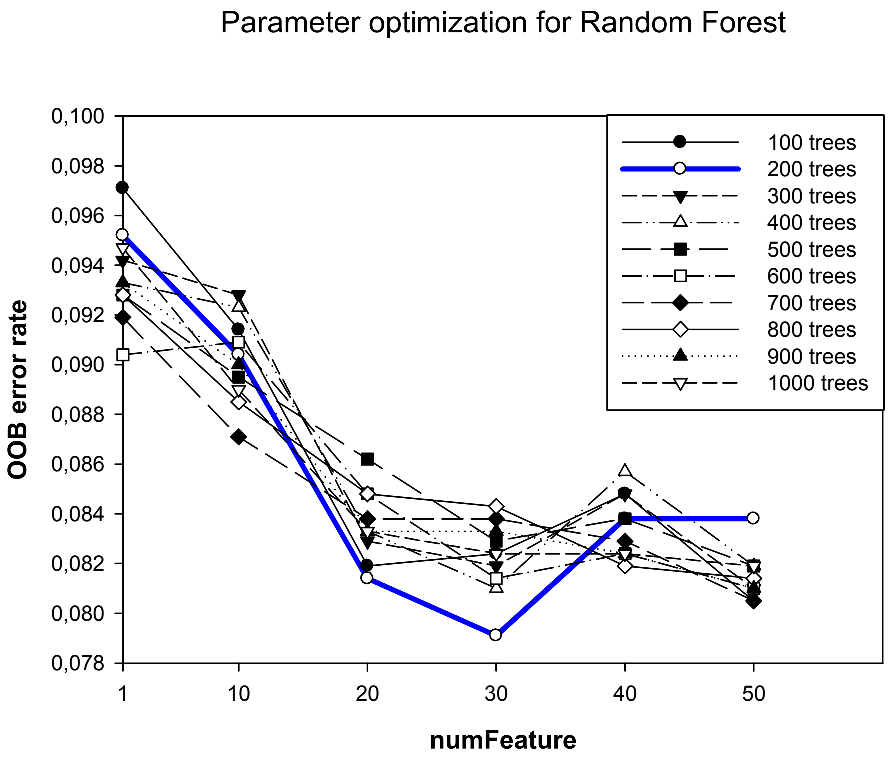

Figure S5. Parameter selection of Random Forest algorithm for interaction prediction trigram model.

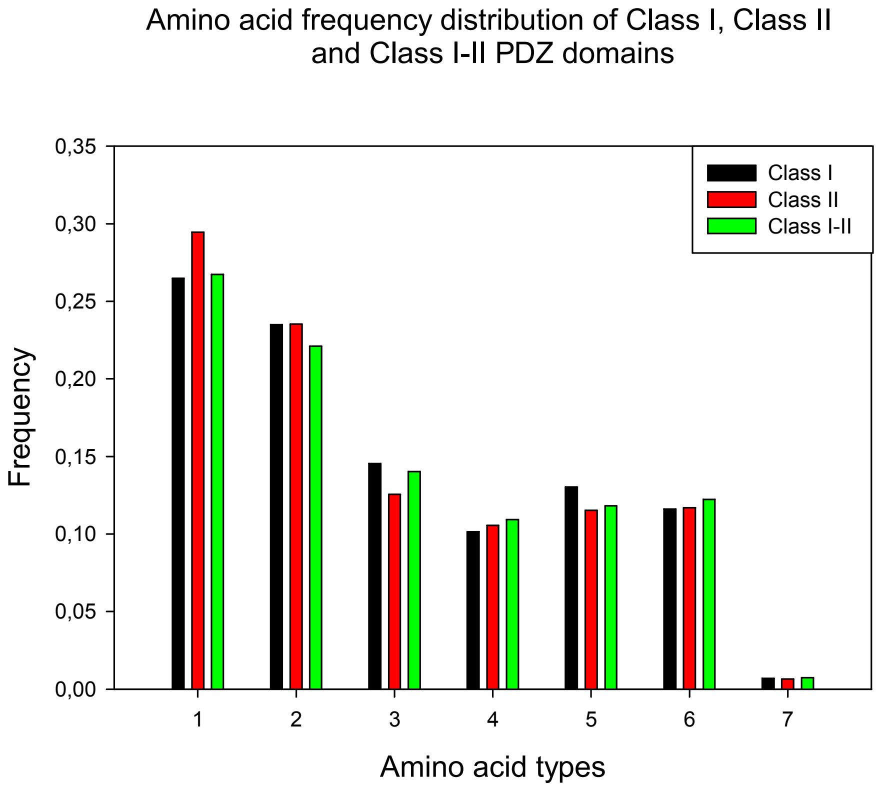

Figure S6. Amino acid frequency distribution of Class I/II/I-II PDZ domains.
